# Distribution of Glycine/GABA Neurons in the Ventromedial Medulla with Descending Spinal Projections and Evidence for an Ascending Glycine/GABA Projection

**DOI:** 10.1371/journal.pone.0035293

**Published:** 2012-04-30

**Authors:** Mehdi Hossaini, Jacqueline A. C. Goos, Somesh K. Kohli, Jan C. Holstege

**Affiliations:** Department of Neuroscience, Erasmus University Medical Center, Rotterdam, The Netherlands; The Research Center of Neurobiology-Neurophysiology of Marseille, France

## Abstract

The ventromedial medulla (VM), subdivided in a rostral (RVM) and a caudal (CVM) part, has a powerful influence on the spinal cord. In this study, we have identified the distribution of glycine and GABA containing neurons in the VM with projections to the cervical spinal cord, the lumbar dorsal horn, and the lumbar ventral horn. For this purpose, we have combined retrograde tracing using fluorescent microspheres with fluorescent in situ hybridization (FISH) for glycine transporter 2 (GlyT2) and GAD67 mRNAs to identify glycinergic and/or GABAergic (Gly/GABA) neurons. Since the results obtained with FISH for GlyT2, GAD67, or GlyT2+GAD67 mRNAs were not significantly different, we concluded that glycine and GABA coexisted in the various projection neurons. After injections in the cervical cord, we found that 29%±1 (SEM) of the retrogradely labeled neurons in the VM were Gly/GABA (RVM: 43%; CVM: 21%). After lumbar dorsal horn injections 31%±3 of the VM neurons were Gly/GABA (RVM: 45%; CVM: 12%), and after lumbar ventral horn injections 25%±2 were Gly/GABA (RVM: 35%; CVM: 17%). In addition, we have identified a novel ascending Gly/GABA pathway originating from neurons in the area around the central canal (CC) throughout the spinal cord and projecting to the RVM, emphasizing the interaction between the ventromedial medulla and the spinal cord. The present study has now firmly established that GABA and glycine are present in many VM neurons that project to the spinal cord. These neurons strongly influence spinal processing, most notably the inhibition of nociceptive transmission.

## Introduction

There are extensive projections from the ventromedial medulla (VM) to the spinal cord, which reach both the dorsal and ventral horns. While in the cat the rostral VM (RVM) projects preferentially to the dorsal horn and the caudal VM (CVM) to the ventral horn, this segregation is less clear in the rat [1], [2]. With respect to the dorsal horn, attention has mainly focused on the role of the RVM in spinal nociception. The RVM, which includes the midline nucleus raphe magnus (RM) and the adjacent reticular formation, is under strong control of the periaqueductal grey (PAG), which produces analgesic effects through the RVM [3]. However, the RVM not only produces inhibition but also facilitation of spinal nociception [4]–[6], which may result in hyperalgesia and allodynia as observed in inflammatory and neuropathic pain models [7]. These findings suggest that there are specific subsets of RVM projection neurons that either inhibit or facilitate spinal nociception. Indeed, electrophysiological studies have shown that so-called ON-cells in the RVM have a net facilitatory effect on nociception, while a second group, designated as OFF-cells, has a net inhibitory effect on nociception [4], [8]. For a long time it was believed that serotonin was responsible for inhibiting spinal nociception [9]–[12] and therefore the most likely transmitter in the OFF cells. However, serotonin was never found in ON- or OFF-cells [13], [14], therefore the neurotransmitters used by the ON- and OFF-cells remain unidentified [4], [5]. Tracing studies, combined with immunohistochemistry for GABA or glutamate decarboxylase (GAD) have shown the existence of GABAergic projections to the dorsal horn [15]–[18] and the ventral horn [19], [20]. However, up to now, the distribution pattern of these spinally projecting GABAergic neuronal somata in the RVM has not been investigated in detail and the location of glycinergic RVM neurons with spinal projections is still unknown.

In order for the RVM to change its activation pattern in response to nociceptive stimuli, it must receive information from the spinal cord [4], [8], [21], [22]. However, anatomical data on direct projections from the spinal cord to the RVM are scarce, and it is assumed that the RVM receives most information from the spinal cord indirectly by projections from the PAG, or lateral paragigantocellular reticular nucleus (LPGi) [23]–[26]. Spinal neurons that project directly to the RVM are found mainly around the central canal [27], of which some contain the neuropeptide enkephalin [28].

In the present study, we have combined retrograde tracing with in situ hybridization for glycine transporter 2 (GlyT2) and GAD67 mRNA to determine the distribution pattern of glycine and/or GABA (Gly/GABA) containing neurons in the VM that project to the cervical and lumbar spinal cord. We found that a substantial percentage of VM neurons that project to the spinal cord contain both GABA and glycine. In addition, we found a novel ascending pathway that contains GABA and glycine. This pathway originates from the area around the central canal throughout the spinal cord, and projects to the RVM.

**Figure 1:**
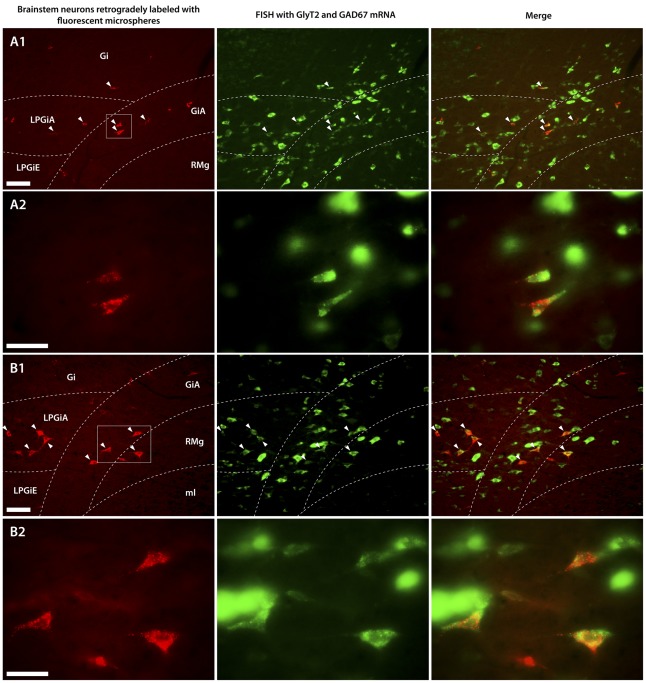
Neurons in the ventromedial medulla that were retrogradely labeled from the spinal cord and neurons labeled for GlyT2 and/or GAD67 mRNAs. Fluorescence micrographs (A1, B1) and their magnifications (A2, B2) showing neurons in the rostral ventromedial medulla (RVM) retrogradely labeled with fluorescent microspheres (red) from the lumbar (L4) dorsal horn (**A**) and the cervical (C5) spinal cord (**B**), and neurons labeled for GlyT2 and/or GAD67 mRNAs (Gly/GABA neurons, green) using fluorescent in situ hybridization (FISH). Arrowheads indicate Gly/GABA neurons that are also retrogradely labeled from the spinal cord. Note that the fluorescent microspheres and the labeling for Gly/GAD67 mRNA are located in the cytoplasm, outside of the neuronal nuclei. The intensity of the fluorescent labeling varied between the labeled neurons, several of which are also out of focus. Gi: gigantocellular reticular nucleus, alpha part (GiA); LPGi: lateral paragigantocellular reticular nucleus, alpha part (LPGiA), external part (LPGiE); Ml: medial lemniscus; RMg: raphe magnus nucleus. Scale bar: 50 µm.

## Materials and Methods

In this study, we used a total of 43 male Wistar rats between 250–300 grams. All animal experiments were approved by the Rotterdam Animal Ethical Committee.

### Tracer Injections

We used FluoSpheres (0.04 µm; molecular Probes, Eugene, OR), consisting of fluorescent polystyrene microspheres for retrograde tracing. For tracer injections in the spinal cord and the brainstem, the rats were kept under general anesthesia with 2% isofluoran in 30%O_2_/70%N_2_O. For injections, a glass micropipette was used, and with each injection between 80 to 100 nanoliter (nl) of tracer was injected using an air pressure device. Further, after each injection the micropipette was left untouched for a period of 3 minutes in order to avoid regurgitation of the injected volume. All injections were made in the left side of the spinal grey matter, or in the midline, left side or both sides of the brainstem. After the operation, the animals were returned to standard housing for a period of 25–28 days.

**Figure 2:**
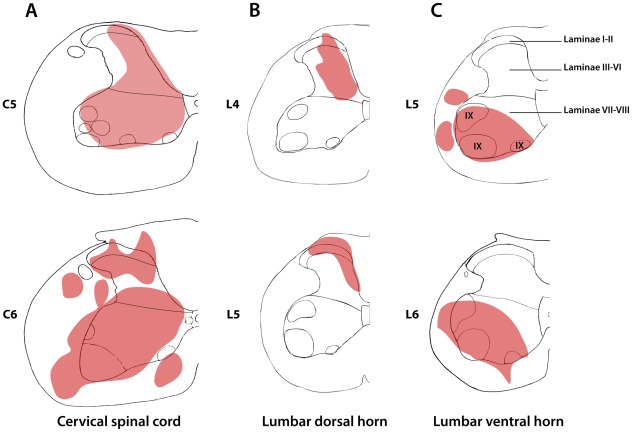
The injection sites of fluorescent microspheres in the rat spinal cord. Drawings illustrating fluorescent microsphere injection sites in the dorsal and ventral cervical (C5–C6) spinal cord (**A**), in the dorsal horn (laminae I to VI) (**B**) and in the ventral horn (laminae VII-X) (**C**) of the lumbar (L4–L6) spinal cord. Note that all injections were confined to one side, and that in some cases fluorescent tracer was also present the adjacent white matter. Roman figures indicate spinal laminae.

### Spinal Cord Injections

For identifying neurons in the RVM that project to the spinal cord, microspheres were injected in the lumbar or cervical enlargement. First, laminectomy was performed at L4–L6 levels (lumbar injections) or at C4–C6 levels (cervical injections) of the spinal cord, producing an open area of approximately 3 mm with a visible dura mater. Next, the animal was fixed using stereotaxic instruments, and the posterior spinal vein was taken as the reference for the midline. In the lumbar spinal cord, injections were made between 0.7 and 0.9 mm lateral to the midline, and in the cervical spinal cord injections were made between 0.5 and 1.5 mm lateral to the midline. For injections in the lumbar dorsal horn (n = 7), the spinal cord was approached dorsally at an angle of 45°, and between 3 to 5 injections were made at 0.5 and 0.9 mm below the surface of the spinal cord. For injections in the ventral horn (n = 5), the spinal cord was approached laterally at an angle of 30°, and between 3 to 5 injections were made at depths of 1.2 and 1.7 mm below the surface of the spinal cord. In the cervical spinal cord (n = 5), we did not differentiate between the dorsal and the ventral horn, and therefore the injections were made at different depths ranging between 0.5 and 1.5 mm.

### Brainstem Injections

Brainstem injections were made in the rostral ventromedial medulla (RVM), encompassing the raphe magnus (RM), gigantocellular reticular nucleus (Gi) and lateral paragigantocellular reticular nuclei (LPGi) (n = 6). For operation, the caudal part of the skull was removed and the caudal end of obex was identified. The injections were made at±1.6 mm rostral to the obex and were limited to the midline (n = 2), between 0.4 and 1.0 mm lateral to the midline on one side (n =  2), or on both sides (n = 2). All injections were made at depths between 4.5 and 5.0 mm below the level of the obex.

**Figure 3:**
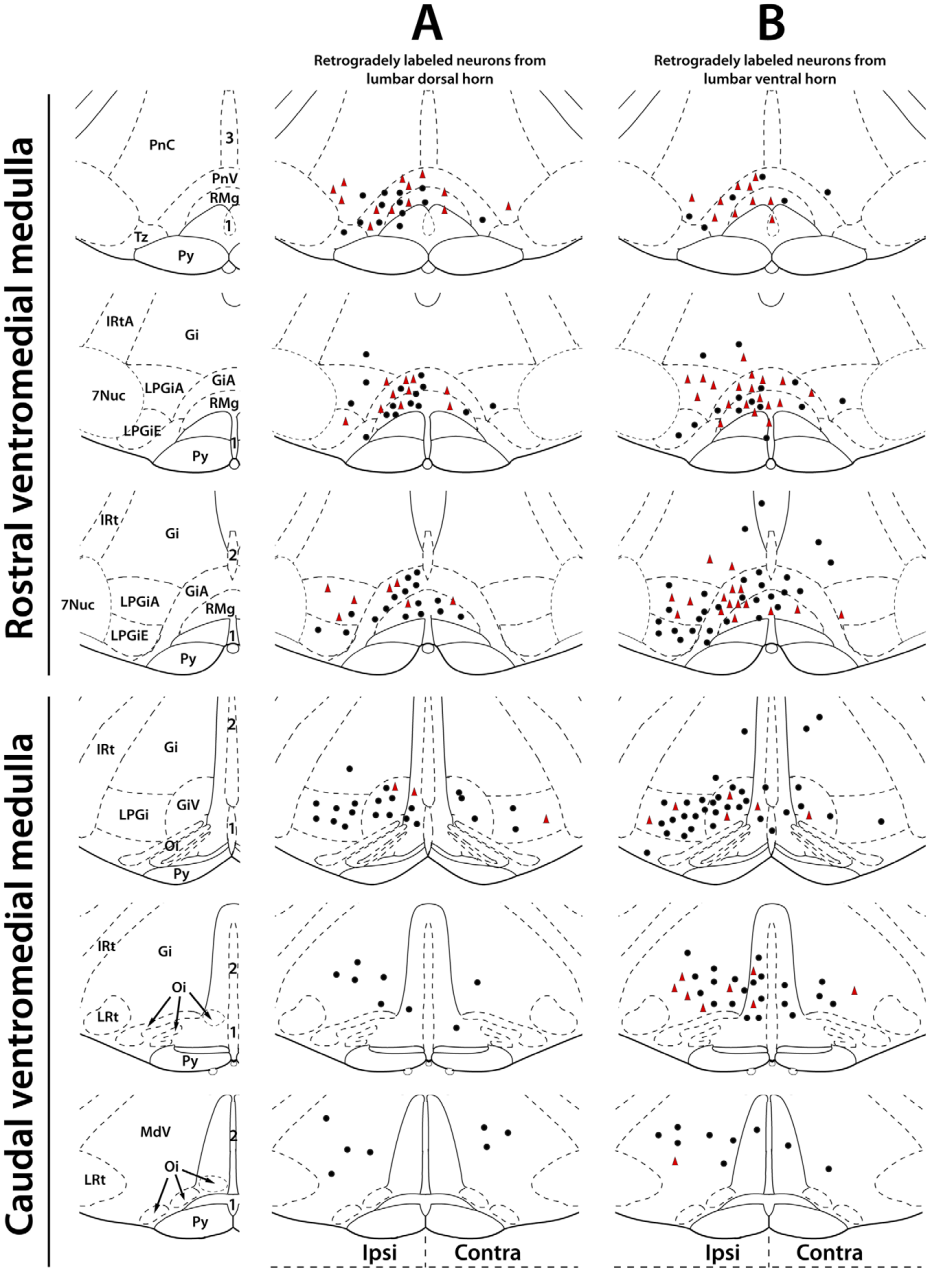
The distribution patterns of neurons in the ventromedial medulla that were retrogradely labeled from the lumbar dorsal or ventral horn. Drawings illustrating representative single brainstem sections of the rostral part of ventromedial medulla (RVM) and the caudal part of ventromedial medulla (CVM). Black filled circles represent neurons retrogradely labeled with fluorescent microspheres from the lumbar dorsal (**A**) or ventral (**B**) horn. Red filled triangles represent retrogradely labeled neurons that also contained GlyT2 and/or GAD67 mRNA (Gly/GABA). Note that the caudal part of the CVM contains fewer retrogradely labeled neurons than the rostral part of the CVM, and that most retrogradely labeled Gly/GABA neurons in both the CVM and RVM are located on the side ipsilateral to the injection. Gi: gigantocellular reticular nucleus, alpha part (GiA); IRt: intermediate reticular nucleus, alpha part (IRtA); LPGi: lateral paragigantocellular reticular nucleus, alpha part (LPGiA), external part (LPGiE); LRt: lateral reticular nucleus; Pn: pontine reticular nucleus, ventral part (PnV) and caudal part (PnC); Py: pyramid tract; Oi: olivary inferior nucleus; RMg: raphe magnus; Tz: nucleus trapezoid body; 7Nuc: nucleus of seventh nerve; 1: raphe pallidus nucleus; 2: raphe obscurus nucleus; 3: raphe interpositus nucleus.

### Tissue Preparation

After survival periods of 25–28 days, the rats received an overdose of sodium pentobarbital, and were then transcardially perfused with 150 ml saline followed by 750 ml of 4% paraformaldehyde (PFA) in phosphate buffer (PB, pH 7.4). Thereafter, the spinal cord and the brainstem were dissected and incubated overnight at 4°C in RNAse free solution consisting of 4% PFA and 30% sucrose in PB. Coronal sections were cut at 30 µm with a freezing microtome, collected in 9 separate jars and stored in a solution consisting of RNAse free PB and formamide (50%/50%) at –20°C. We did not use glycerol for storage, since it appeared to reduce the fluorescence intensity of the beads over time. This was not observed when sections were stored in PB/formamide solution.

**Figure 4:**
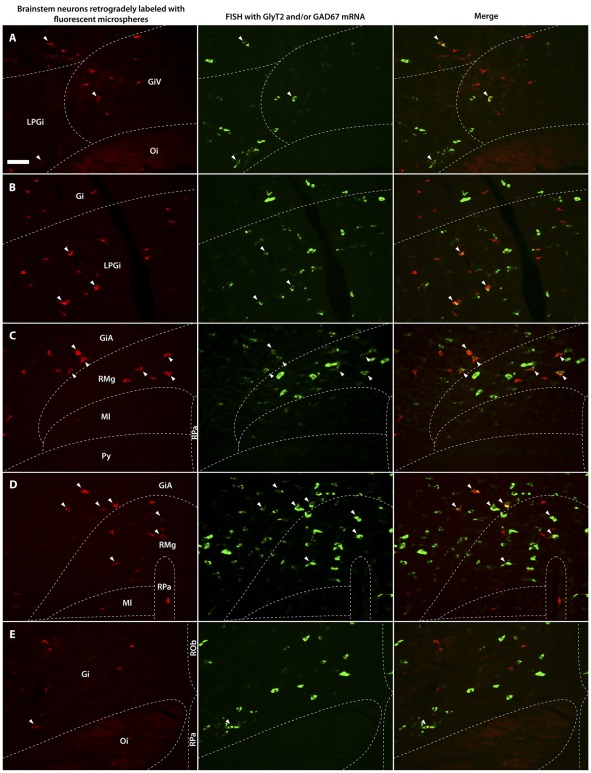
Neurons in the ventromedial medulla that were retrogradely labeled from the spinal cord and neurons labeled for GlyT2 and/or GAD67 mRNAs. Fluorescence micrographs showing neurons in the ventromedial medulla retrogradely labeled with fluorescent microspheres (red) from the spinal cord and neurons labeled for GlyT2 and/or GAD67 mRNA (green) using FISH. Arrowheads indicate GABAergic (GAD67 mRNA containing) neurons in the caudal ventromedial medulla (CVM) retrogradely labeled from the cervical spinal cord (**A**); glycinergic (GlyT2 mRNA containing) neurons in the rostral ventromedial medulla (RVM) retrogradely labeled from the cervical spinal cord (**B**); GlyT2 and/or GAD67 mRNA containing neurons (Gly/GABA) in the RVM retrogradely labeled from the lumbar dorsal horn (**C**); Gly/GABA neurons in the RVM retrogradely labeled from the lumbar ventral horn (**D**); Gly/GABA neurons in the CVM retrogradely labeled from the lumbar ventral horn (**E**). Scale bar = 50 µm.

**Figure 5:**
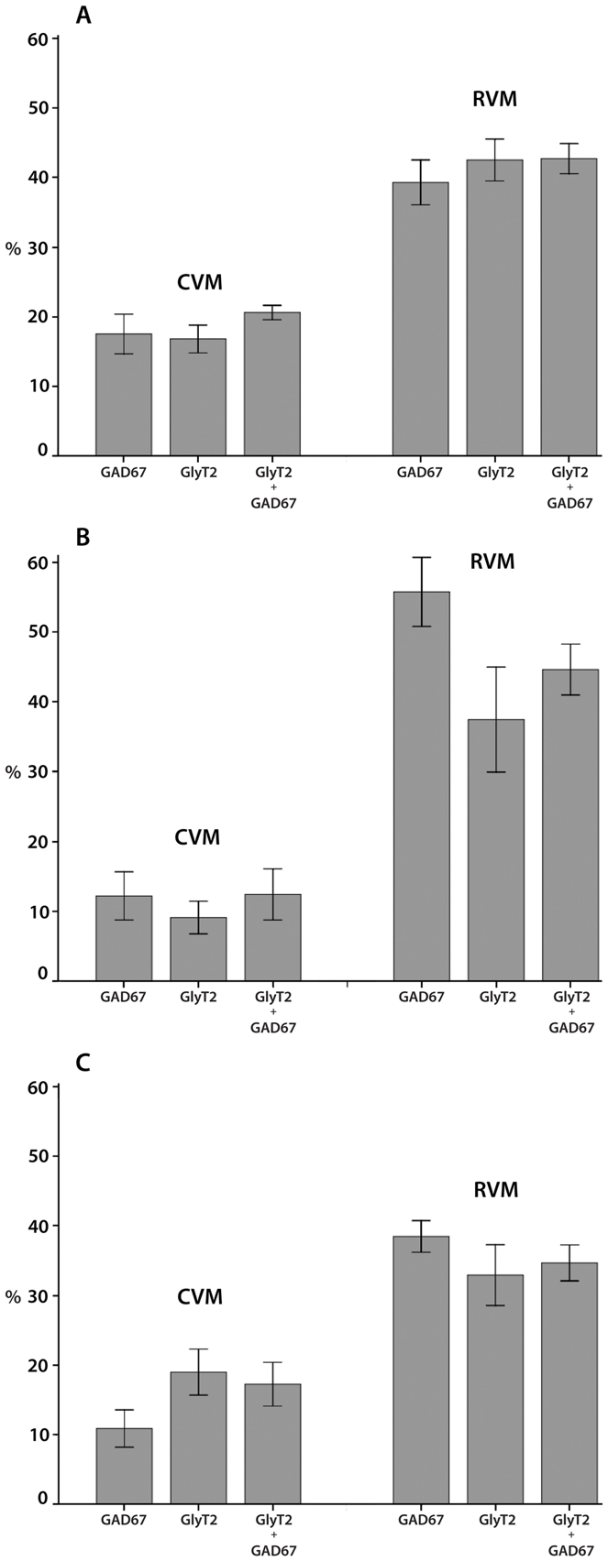
The percentages of neurons in the ventromedial medulla that were retrogradely labeled from the spinal cord and contained GlyT2 and/or GAD67 mRNAs. Histograms showing the percentages of retrogradely labeled neurons in the ventromedial medulla that contained GAD67, GlyT2, and GlyT2 and/or GAD67 mRNAs after fluorescent microsphere injections in the cervical spinal cord (**A**), in the lumbar dorsal horn (**B**) and in the lumbar ventral horn (**C**). The percentages are shown separately for the caudal ventromedial medulla (CVM) and rostral ventromedial medulla (RVM) and were always significantly lower in the CVM than in the RVM (*p*<0.05). Differences between the various percentages within the CVM and the RVM were never significant (*p*>0.1).

### Fluorescent in Situ Hybridization

Fluorescent in situ hybridization (FISH) was performed using the protocol described previously [29], [30]. We used cDNA templates (GlyT2 cDNA: 3.1 kb; a generous gift from Dr. N. Nelson, Tel Aviv University; GAD67 cDNA: 3.2 kb; a generous gift from Dr. A.J. Tobin and N. Tillakaratne, PhD, UCLA) to produce the GlyT2 and GAD67 mRNA probes in the presence of Digoxigenin-labeled 11-UTP. After riboprobe hybridization, we applied immunohistochemistry (IHC) with a sheep anti-digoxigenin polyclonal antibody (Roche) to identify digoxigenin (4°C, 48hrs). Subsequently, the primary antibody was identified with biotinylated rabbit-anti-goat antibody (Vector) (room temperature (RT), 90 min), followed by incubation with Avidin Biotin Complex (ABC) tagged with horseradish peroxidase (HRP) (RT, 90 min). Thereafter, a tyramide amplification procedure was performed by reacting HRP with H_2_O_2_ and a self-made FITC tyramide as described previously [31]. Finally, sections were washed in PB, randomly mounted on slides and coverslipped with Vectashield (Vector).

**Figure 6:**
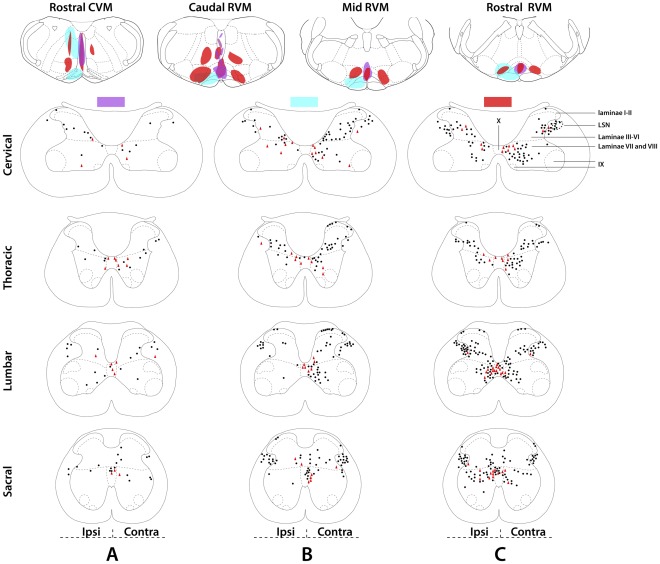
The distribution patterns of neurons in the spinal cord that were retrogradely labeled from the rostral ventromedial medulla. Schematic drawings showing the brainstems of three rats with fluorescent microsphere injections (blue, purple and red) and the corresponding spinal cords with the distribution of retrogradely labeled neurons (filled black circles), and retrogradely labeled spinal neurons also containing GlyT2 and/or GAD67 mRNA (red triangles). Each segmental drawing, i.e. cervical (C4–C8), thoracic (T1–T3 and T10–T12), lumbar (L1–L6) and sacral (S1–S4), shows the cumulative results obtained in 8-10 sections. Tracer injections were aimed at rostral ventromedial medulla (RVM) with injections confined to the midline (purple), to one side only (blue), or both sides of the RVM (red). Note that tracer injections limited to the midline yield fewer retrogradely labeled neurons in the spinal cord (purple), and injections limited to one side of the brainstem, yield more retrogradely labeled neurons on the contralateral side of the spinal cord (blue as compared to purple and red). Roman figures indicate spinal laminae. LSN: lateral spinal nucleus.

### Analyzing labeled Neurons

In rats with tracer injections in the cervical or lumbar spinal cord, sections from the caudal medulla at three levels that included the inferior olive (Oi) nucleus (2–3 sections per level), and sections at three levels ranging from caudal to rostral RVM were analyzed (2–3 sections per level). In rats with tracer injections in the RVM, we analyzed sections from the cervical, upper and lower parts of the thoracic, lumbar and sacral segments. Sections for analysis were chosen by starting in the first row of the randomly mounted sections and searching for sections from the appropriate segmental level (C4 to T5 for cervical and upper thoracic segments; T10 to S4 for lower thoracic, lumbar and sacral segments). Per rat, 10–12 sections for cervical and upper thoracic segments, and 12–14 sections for lower thoracic, lumbar and sacral segments were analyzed in a Leica fluorescent microscope with a FITC and/or Cy3 filter.

**Figure 7:**
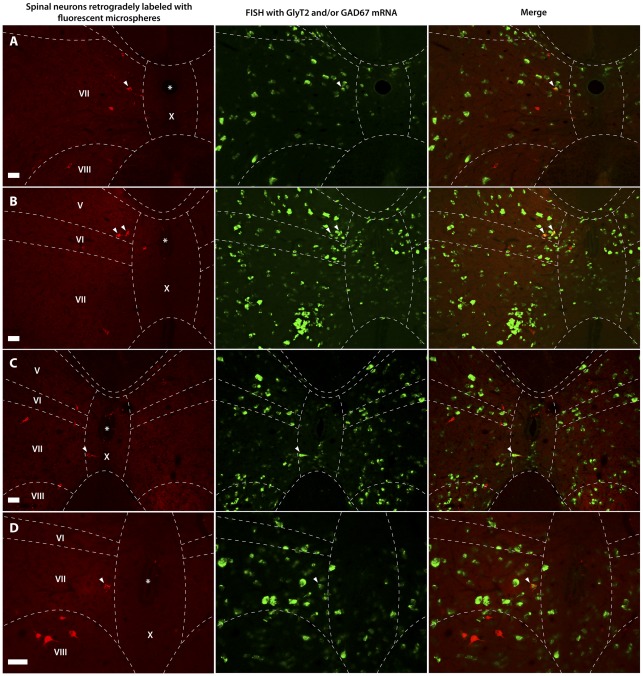
Spinal neurons retrogradely labeled from the rostral ventromedial medulla, and spinal neurons labeled for GlyT2 and/or GAD67 mRNAs. Fluorescence micrographs showing spinal neurons (red) retrogradely labeled with fluorescent microspheres from the rostral ventromedial medulla (RVM) and spinal neurons (green) containing GlyT2 and/or GAD67 mRNA (glycinergic and/or GABAergic neurons) identified with FISH. Arrowheads indicate spinal neurons in the area around the central canal, which are retrogradely labeled from the RVM and are also labeled for GlyT2 mRNA in segment C5 (**A**) and for GlyT2 and/or GAD67 mRNAs in segments L5 (C) and L6 (**B and D**). Scale bar = 50 µm.

In order to investigate colocalization of the FluoSpheres beads (apparent as red particles using a Cy3 filter) with GlyT2/GAD67 mRNAs (FITC) staining, first labeling for the tracer (red particles) was determined in a 40x objective. A neuron was counted as positive for tracer labeling if at least ten bright red particles were present in the soma. Then, in the same focus field, we determined whether there was labeling for GlyT2/GAD67 mRNAs present in the cytoplasm. If in the same focus field, tracer labeling was present together with somatic GlyT2/GAD67 mRNAs staining, the neuron was counted as a double-labeled neuron. Single tracer labeled neurons and tracer labeled neurons that contained GlyT2/GAD67 were plotted by hand in an illustrated representation of the appropriate segmental level. In the illustrations used for spinal cord sections, the grey matter was divided in 3 areas (area 1: laminae I and II; area 2: laminae III-VI; area 3: laminae VII-X) according to the laminar distribution in the rat [32], [33]. For brainstem sections, we used illustrations of brainstem sections according to the rat brain atlas [34]. Per rat, the number of neurons labeled with tracer only, with tracer/GlyT2, tracer/GAD67 or tracer/GlyT2/GAD67 was determined for the RVM, the CVM or the different spinal cord areas, and for each area the percentages of tracer labeled neurons that were double labeled were calculated. The results obtained per rat for each of the various areas and injection sites were averaged. Errors in the variations were assessed as standard error of the mean (SEM). The unpaired *t*-test or one-way ANOVA with a Bonferroni post-hoc test was performed for the various statistical comparisons and *p*<0.05 was taken as significant.

## Results

### General Observations

Inhibitory neurons, i.e. neurons that use either glycine or GABA or both transmitters, were labeled by means of fluorescent in situ hybridization (FISH). We used FISH with probes for GlyT2 mRNA (for identifying glycinergic neurons), or GAD67 mRNA (for identifying GABAergic neurons), or with a mixture of probes for GlyT2 and GAD67 mRNAs (GlyT2/GAD67) for identifying glycinergic and/or GABAergic (Gly/GABA) neurons (Fig. 1). FISH was applied on sections from the brainstem and the spinal cord, resulting in greenish-labeled neuronal somata of various sizes. The distribution patterns of the labeled neurons, i.e. glycinergic and/or GABAergic, in the spinal cord and the brainstem were in general accordance with previous findings [35]–[38].

After injection of fluorescent microspheres in the spinal cord and brainstem, we found retrogradely labeled neurons of various sizes in the brainstem and spinal cord, respectively. The retrogradely labeled neurons contained bright red particles, which represented the retrogradely transported microspheres, which were present in the somata and primary dendrites of the labeled neurons (Fig. 1). The number of red particles varied considerably between the labeled neurons.

### Neurons in the Ventromedial Medulla Retrogradely Labeled from the Spinal Cord

#### Injection sites

In the cervical spinal cord, several injections with fluorescent microspheres were made unilaterally at the C5–C6 level. The injections included nearly the entire grey matter and parts of the adjoining white matter (Fig. 2A). For injections in the lumbar spinal cord, we injected fluorescent microspheres in the dorsal or the ventral horn of the lumbar segments L4–L6. With respect to the dorsal horn (Fig. 2B), injection sites included laminae I to VI, and in some cases the dorsal and/or lateral parts of the white matter. The injections in the ventral horn (Fig. 2C) included laminae VII to IX, and parts of the lateral and ventral white matter. Since the latter injections were made using a lateral approach, they never included any part of the dorsal horn or its adjacent white matter.

#### Overview of the results

We analyzed the distribution of retrogradely labeled neurons in the ventromedial medulla, which consists of the rostral ventromedial medulla (RVM) and the caudal ventromedial medulla (CVM). The RVM includes the nucleus raphe magnus (RM), raphe pallidus (RP), gigantocellular reticular nucleus (Gi), part alpha of Gi nucleus (GiA), part alpha of lateral paragigantocellular (LPGi) nucleus (LPGiA), and the external part of LPGi nucleus (LPGiE). The CVM includes the RP, raphe obscurus (ROb), the ventral part of the medullary reticular nucleus (MdV), Gi nucleus, the ventral part of Gi (GiV), and LPGi nucleus, at levels ranging from the most caudal to the most rostral part of the olivary inferior nucleus (Oi). We first determined, for each of the injection sites, the overall percentages of retrogradely labeled neurons in the ventromedial medulla that were also labeled for GlyT2 and/or GAD67 mRNA (Gly/GABA neurons). After injections in the cervical spinal cord 29%±1 (SEM) of the retrogradely labeled neurons were Gly/GABA. After injections in the lumbar dorsal horn and ventral horn (Fig. 3), these percentages were 31%±3 and 25%±2, respectively. We found no significant differences in the percentages between neurons retrogradely labeled from cervical, lumbar dorsal and ventral horns. In the next sections, the results are presented in more detail.

#### Neurons in the ventromedial medulla retrogradely labeled from the cervical spinal cord

After injections in the C5-C6 spinal cord, which encompassed most of the dorsal and ventral horns, a total of 3257 retrogradely labeled neurons was analyzed for the RVM and CVM separately (Fig. 4A, B). Most retrogradely labeled neurons were found on the side ipsilateral to the injection (RVM: 87%±3; CVM: 75%±2). The percentages of retrogradely labeled neurons (ipsi+contra) that contained GlyT2, GAD67, or GlyT2/GAD67 mRNA are shown in Fig. 5A. These percentages were always significantly lower in the CVM than in the RVM (*p*<0.005, ANOVA). Within the RVM and the CVM there were no significant differences between the various percentages (*p*>0.1, ANOVA, Fig. 5A) nor between the ipsilateral and contralateral sides (*p*>0.1, ANOVA; data not shown).

#### Neurons in the ventromedial medulla retrogradely labeled from the lumbar dorsal horn

After injections in the L4–L6 dorsal horn, we counted in total 2334 retrogradely labeled neurons (Figs. 3A and 4C). The majority of the retrogradely labeled neurons was found ipsilateral to the injection (RVM: 77%±2; CVM: 67%±10). Fig. 5B shows that in the RVM the percentages of retrogradely labeled neurons (ipsi+contra) that contained GAD67 was higher than the percentages obtained for GlyT2 and GlyT2/GAD67 mRNA. However, these differences were not significant (*p*>0.5, ANOVA). In the CVM, there were also no significant differences between the various percentages (*p*>0.1, ANOVA), but they were always significantly lower than in the RVM (*p*<0.05, ANOVA). With respect to the various percentages obtained for the ipsi and contralateral side, there were no significant differences (*p*>0.05, ANOVA; data not shown).

#### Neurons in the ventromedial medulla retrogradely labeled from the lumbar ventral horn

After injections in the L4–L6 ventral horn, a total of 3640 retrogradely labeled neurons was analyzed (Figs. 3B and 4D, E). Most retrogradely labeled neurons were found on the side ipsilateral to the injection (RVM: 76%±3; CVM: 51%±16). The percentages of retrogradely labeled neurons (ipsi+contra) that contained GlyT2, GAD67, or GlyT2/GAD67 mRNA are depicted in Fig. 5C and were always significantly lower in the CVM than in the RVM (*p*<0.05, ANOVA). Within the RVM and the CVM, there were no significant differences between the various percentages (*p*>0.1, ANOVA, Fig. 5C) nor between the ipsilateral and contralateral sides (*p*>0.1, ANOVA; data not shown), except that in the CVM on the ipsilateral side 8%±1 of the retrogradely labeled neurons also contained GAD67, while significantly higher percentages contained GlyT2 (19%±3) and GlyT2/GAD67 (19%±3) mRNAs (*p*<0.05, ANOVA). This difference was not found on the contralateral side.

### Neurons in the Spinal Cord Retrogradely Labeled from the Ventromedial Medulla

#### Injection sites

In this experiment, we analyzed retrogradely labeled neurons in the spinal cord after injections of fluorescent microspheres in the RVM area. We found that many of the injection areas included the RM nucleus, the RP nucleus, and the medial part of the LPGi nucleus and the Gi nucleus, and parts of the pyramidal tract. After injections in the RVM that were limited to the midline, fewer retrogradely labeled neurons were found in the spinal cord (Fig. 6A). Further, when RVM tracer injections were limited to one side only, we found that the majority of retrogradely labeled neurons in the spinal cord were located on the contralateral side (Fig. 6B).

#### Spinal distribution of the retrogradely labeled neurons

Our results showed that the majority of the retrogradely labeled neurons was located in the area around the central canal (laminae X, and the adjoining medial parts of laminae IV, V, VI, and VII), in the lateral part of lamina V and the adjoining white matter including the lateral spinal nucleus (LSN) (Fig. 6). Some retrogradely labeled neurons were also present in lamina I. At all levels of the spinal cord that were examined, i.e. the cervical (C4–C8), upper thoracic (T1–T5), low thoracic (T10–T12), lumbar (L1–L6) and sacral (S1–S4) segments, retrogradely labeled neurons that contained GlyT2/GAD67 mRNA were predominantly located in the area around the central canal (Fig. 7), while very few double-labeled neurons were present in the lateral parts of lamina V and the LSN (Fig. 6). On average, 14%±2 of the retrogradely labeled neurons in the grey mater also contained GAD67 mRNA, 15%±1 contained GlyT2 and 16%±1 contained GlyT2/GAD67 mRNA. These percentages were not significantly different (*p*>0.5, ANOVA). Furthermore, we did not find significant differences between various segmental areas in the spinal cord (*p*>0.5, ANOVA, data not shown).

## Discussion

In the present study, we have identified projection neurons in the ventromedial medulla and the spinal cord using fluorescent microspheres as a retrograde tracer. This technique is highly efficient for retrograde tracing as it is taken up by terminals rather than by intact passing fibers [39]. The retrograde tracing was combined with fluorescent in situ hybridization (FISH) using probes for GlyT2 mRNA [36], [40], GAD67 mRNA [41], [42] and a combination of these probes to separately identify glycinergic, GABAergic and glycine and/or GABA containing (Gly/GABA) neurons, respectively as described in our previous studies [29], [30]. For each of the various injection sites (i.e. the cervical spinal cord, the lumbar dorsal horn, the lumbar ventral horn and the RVM), the percentages of the retrogradely neurons that were glycinergic, GABAergic or Gly/GABA were not significantly different. We therefore concluded that GABA and glycine coexist in the large majority, if not all, of the retrogradely labeled neurons containing either glycine or GABA, based on the reasoning that otherwise the percentage of Gly/GABA neurons would have been significantly higher. This conclusion is also in line with the extensive colocalization of GABA and glycine in other regions of the brainstem [37] and in the spinal cord [43], [44], and the co-release of GABA and glycine that was identified in lamina I of the spinal cord [45]. Although the functional significance of the co-release of GABA and glycine is still unclear, it seems most likely that the bulk of fast synaptic inhibition is produced by glycine, while GABA is involved primarily in negative feedback through presynaptic and extrasynaptic sites [46]. In view of the extensive colocalization of GABA and glycine, we will refer to these neurons as Gly/GABA neurons in the remainder of the discussion.

We found that 29% of the neurons in the ventromedial medulla projecting to the cervical cord were Gly/GABA, while these percentages were 31% and 25% for the projections to the dorsal and ventral horn, respectively. The high similarity of these percentages may be explained by the high level of collateralizations that was found in the spinal projections from the ventromedial medulla to the rostral and caudal parts of the spinal cord, as well as the dorsal and ventral horns [2]. In addition to the descending Gly/GABA projections, we also showed that there are Gly/GABA neurons, predominantly located in the area around the central canal throughout the spinal cord, with ascending projections to the RVM. This finding is the first evidence for a fast inhibitory spinal system, projecting to supraspinal levels.

### Gly/GABA Projections from the Ventromedial Medulla to the Dorsal Horn

For the identification of Gly/GABA neurons in the ventromedial medulla that project to the dorsal horn, we have focused on the lumbar cord, but in view of the high level of collateralization in the ventromedial medullary projections to the spinal cord [2], they are likely to hold true also for the other spinal segments. The majority of the retrogradely labeled neurons were present in the RVM and the rostral part of the CVM, which is in general agreement with previous studies [2], [47]–[49]. The percentage of retrogradely labeled neurons that were Gly/GABA was higher in the RVM (45%) than in the CVM (12%), showing that the majority of the Gly/GABA projection neurons are located in the RVM. The percentage of GABAergic neurons (56%) in the RVM was higher than that of the glycinergic neurons (37%). Although this difference was not significant, it may indicate that some of the RVM projections to the dorsal horn contain only GABA and not glycine.

Previous studies, using retrograde tracing combined with GABA immunohistochemistry (IHC) [18], GAD IHC [16], [17] or ISH [50], have shown that about 10–30% of the neurons retrogradely labeled from the spinal cord were GABAergic. Anterograde tracing from the RVM combined with GABA IHC at the ultrastructural level [15] showed that 95% of the RVM derived terminals in various laminae of the dorsal horn were GABAergic (ascribed to the fact that the anterograde tracer was taken up specifically by GABAergic neurons), with 12% of these terminals also containing glycine. This latter finding is, so far, the only anatomical evidence for a glycinergic projection to the dorsal horn. Our findings now provide direct evidence for a glycinergic projection to the dorsal horn, which is likely contained within the GABAergic projection.

The RVM may facilitate or inhibit spinal nociception depending on the type and the temporal-spatial characteristics of the nociceptive stimulus [5], [51]. The facilitatory effect of the RVM is exerted by the physiologically characterized ON-cells, while the inhibitory effect is mainly produced by the physiologically characterized OFF-cells [3], [5], [52], [53]. In the present study, we have shown that about 45% of the neurons projecting to the spinal dorsal horn are Gly/GABA, suggesting that glycine and GABA are contained within the OFF-cells. Indeed, several studies have shown the relation of RVM stimulation with antinociception exerted by GABAergic and glycinergic neurotransmission in the dorsal horn [54]–[57] and recently, a technically demanding study on individual physiologically identified neurons [58], showed that some OFF-cells were immunoreactive for GABA, although some ON cells were positive as well. Taken together, there is strong evidence that the GABAergic neurons in the ventromedial medulla, many of which also contain glycine, are responsible for the RVM inhibition of spinal nociception.

### Gly/GABA Projections from the Ventromedial Medulla to the Lumbar Ventral Horn

Microsphere injections in the lumbar ventral horn, which never involved the dorsal horn, resulted in about equal numbers of retrogradely labeled neurons in the RVM and the CVM as also found in previous studies [2], [49]. We found that several of the retrogradely labeled neurons were Gly/GABA (RVM 35%, CVM 17%). The only studies on Gly/GABA projections to the ventral horn were performed in our lab previously, using anterograde tracing from the ventromedial medulla to spinal motoneuronal cell groups combined with GABA [19] or glycine [20] immunohistochemistry at the ultrastructural level. These studies showed that 40% of the descending projections were GABAergic and 15% were glycinergic. Our present retrograde findings, which also include projections to the spinal intermediate zone, strongly indicate that GABA and glycine are colocalized in the descending projections to the ventral horn, suggesting that the finding of 15% being glycinergic may be an underestimation. We found that a relatively low percentage of spinally projecting neurons in the CVM were Gly/GABA, which fits well with the recent finding [59] that 61% of these neurons contain the vesicular glutamate transporter 2, indicating they are glutamatergic.

With respect to the functional meaning of the descending Gly/GABA projections from the ventromedial medulla, attention has focused primarily on the question whether these Gly/GABA projections are involved in producing muscle atonia during REM sleep [1], [60] or whether glutamatergic projections from the ventromedial medulla induce motoneuronal inhibition through inhibitory spinal interneurons. However, it seems likely that the descending Gly/GABA projections may also be involved in regulating motor activity during wakefulness, by modulating somatomotor and visceromotor functions [61], [62].

### Gly/GABA Projections from the Spinal Cord to the RVM

For identifying the presence of a Gly/GABA projection from the spinal cord to the RVM, several injections were made in the RVM area. While the injection sites varied in size, the resulting distribution pattern of retrogradely labeled neurons in the spinal cord was always similar and in line with previous studies using retrograde tracing from the RVM [28], [63]. We found that many neurons projecting to the RVM are located in the area around the central canal (CC), and that about 15% of these projections neurons are Gly/GABA. This pathway constitutes the only projection from the spinal cord to a supraspinal structure (i.e. the RVM area) with the fast inhibitory transmitters GABA and glycine. Anterograde tracing from the CC area confirmed the presence of direct projections, albeit limited, to the RVM [27]. Many neurons in the CC area have been identified to respond to innocuous and noxious stimuli from the periphery and the viscera [64]–[68], indicating an important role of the CC area in the processing of sensory and especially visceral nociceptive information. Whether the Gly/GABA neurons in the CC area respond to peripheral and visceral noxious stimuli and whether the ascending inhibitory projections are involved in inhibiting the spinal projection neurons in the RVM is presently unclear.

### Conclusions

The present study shows that many neurons in the ventromedial medulla that project to the spinal cord contain both GABA and glycine. It seems likely that the inhibitory projections to the dorsal horn are involved in the inhibition of pain transmission, while the projections to the ventral horn are likely involved in modulating motor control during sleep and wakefulness. We also have shown the existence of Gly/GABA containing spinal projection neurons, originating mainly in the CC area and terminating in the RVM area, emphasizing the complicated interaction between the ventromedial medulla and the spinal cord. The extensive projections of the ventromedial medulla to the spinal cord makes it a very powerful system, possibly controlling the proper homeostatic functioning of the entire organism [12]. Our study has now definitely established that a substantial part of this system uses glycine and GABA to exert its functions.
